# Recruitment barriers in a randomized controlled trial from the physicians' perspective – A postal survey

**DOI:** 10.1186/1471-2288-9-14

**Published:** 2009-03-02

**Authors:** Anne Spaar, Martin Frey, Alexander Turk, Werner Karrer, Milo A Puhan

**Affiliations:** 1 Horten Centre for patient-oriented research and knowledge transfer, University of Zurich, Switzerland; 2 Klinik Barmelweid, Barmelweid, Switzerland; 3 Zuercher Hoehenklinik Wald, Wald, Switzerland; 4 Luzerner Hoehenklinik Montana, Montana, Switzerland; 5 Department of Epidemiology, Johns Hopkins Bloomberg School of Public Health, Baltimore, Maryland, USA

## Abstract

**Background:**

The feasibility of randomized trials often depends on successful patient recruitment. Although numerous recruitment barriers have been identified it is unclear which of them complicate recruitment most. Also, most surveys have focused on the patients' perspective of recruitment barriers whereas the perspective of recruiting physicians has received less attention. Therefore, our aim was to conduct a postal survey among recruiting physicians of a multi-center trial to weigh barriers according to their impact on recruitment.

**Methods:**

We identified any potential recruitment barriers from the literature and from our own experience with a multi-center trial of respiratory rehabilitation in patients with chronic obstructive pulmonary disease. We developed and pilot-tested a self-administered questionnaire where recruiting physicians were asked to express their agreement with statements about recruitment barriers on a Likert-type scale from 1 (full agreement with statement = very substantial recruitment barrier) to 7 (no agreement with statement = no recruitment barrier).

**Results:**

38 of 55 recruiting physicians returned questionnaires (69% response rate), of which 35 could be analyzed (64% useable response rate). Recruiting physicians reported that "time constraints" (median agreement of 3, interquartile range 2–5) had the most negative impact on recruitment followed by "difficulties including identified eligible patients" (median agreement of 5, IQR 3–6). Other barriers such as "trial design barriers", "lack of access to treatment", "individual barriers of recruiting physicians" or "insufficient training of recruiting physicians" were perceived to have little or no impact on patient recruitment.

**Conclusion:**

Physicians perceived time constraints as the most relevant recruitment barrier in a randomized trial. To overcome recruitment barriers interventions, that are affordable for both industry- and investigator-driven trials, need to be developed and tested in randomized trials.

**Trial registration:**

ISRCTN84612310

## Background

Patient recruitment is one of the greatest barriers in the conduct of randomized controlled trials (RCT). Trials from all medical specialties face problems in recruiting patients. Barriers hampering recruitment can be due to patient preferences, eligibility criteria, organizational problems or other factors [1-15]. There is agreement that every effort should be made during the planning stage and during the trial to identify potential recruitment barriers [16] and to choose adequate preventative measures to minimize them [4,5,8,9,16-18]. However, measures addressing potential recruitment barriers require substantial financial and time resources and, often, it is not possible to take all of them. As a consequence of limited resources it is essential to focus on the most important barriers and to choose effective measures to minimize them if available.

In recent years a number of studies aimed at identifying barriers in patient recruitment as perceived by patients, health care providers and investigators. Systematic reviews summarizing these studies found that certain barriers were identified consistently across studies including *system-related barriers *(for example lack of study staff), *individual barriers *(for example treatment preferences of health care providers) and *trial-design-related barriers *(for example restrictive eligibility criteria) [3,6,7,15,16]. These studies are very informative to have an overview of any type of recruitment barrier that might be present. They had, however, two major limitations. First, their methodological quality was low [16] and second, the studies offered no weighting of the importance of the identified recruitment barriers. As discussed above, it is important to prioritize those barriers that have the largest impact on recruitment and where effective measures to counteract them are available. To inform investigators, which barriers should be prioritized when recruitment problems are experienced or are to be expected, we conducted a postal survey among physicians who recruit patients into a multi-center RCT with substantial recruitment problems in order to weigh the importance of a number of known recruitment barriers.

## Methods

### Study design

We conducted a postal survey among recruiting physicians of a respiratory rehabilitation trial enrolling patients with Chronic Obstructive Pulmonary Disease (COPD). To optimize the return rate of the questionnaires, we informed the recruiting physicians in our quarterly electronic newsletter 2 weeks before mailing the questionnaires and reminded participants once electronically two weeks after sending the questionnaire to them.

### Description of the RCT

The trial is a multi-centre RCT on respiratory rehabilitation in COPD patients in Switzerland (trial registration ISRCTN84612310 on http://www.controlled-trials.com/isrctn/). Patients are randomly assigned to either early (within two weeks after completion of the exacerbation treatment) or delayed respiratory rehabilitation after six month in a stable pulmonary state. In some countries such as Canada, the USA or France patients are usually referred to respiratory rehabilitation only when patients are in a stable state. Advantages of this approach are that patients may adhere better to the training program. In other countries including Switzerland, Germany or Austria patients are traditionally sent to early rehabilitation, which may offer advantages in respect to effective patient education ("window of opportunity" [19]) and continuity of the post-exacerbation management. Both approaches proved to be effective in meta-analyses of RCTs comparing early and late rehabilitation with usual care (no rehabilitation) [20,21]. It is, however, unclear whether one approach might be superior over the other. The primary outcome of the trial is exacerbation requiring medical treatment (event-based definition) and secondary outcomes include health-related quality of life, mortality and costs. Patients eligible for recruitment have COPD GOLD stage II-IV and a history of repeated exacerbations. From September 2006 to January 2009, only 37 patients were randomized, which is still far from the target sample size of 280. The RCT was approved by all regional ethic committees and patients provide written informed consent. For the sub-study presented in this paper it was not necessary to obtain additional ethical approval.

### Participants

40 physicians from 24 hospitals and 15 physicians from 13 private practices are involved in the recruitment process. They assess patients for eligibility (time requirement of <5 minutes), inform patients about the study and ask them for informed consent (20–30 minutes) and collect the baseline data (20–30 minutes). Incentives to enroll patients include group co-authorship and 200 Swiss Francs per enrolled patient (around 120 Euro or 190 $). We invited all recruiting physicians to participate in this survey to avoid any selection bias [16]. Most of the physicians work in the German-speaking part of Switzerland (rural and urban areas) whereas few doctors work in the French-speaking part of Switzerland. The recruiting physicians hold different specializations, the most frequent being General Internal Medicine and Respiratory Medicine. We instructed each recruiting physician personally about the recruitment process in one to three meetings and provided the study material in printed or electronic form as they preferred. The study coordinator (AS) and a study nurse are available to support the recruiting physicians in any aspect related to the recruitment process.

### Questionnaire development and pilot testing

The questionnaire had two parts. In the first part we asked the participants about age, work place setting, specialization, year of medical licensure and asked participants about their personal recruitment success (number of eligible patients, number of eligible patients asked to participate and number of eligible patients who refused to participate). In the second part we asked participants about recruitment barriers. The questions about recruitment barriers were developed as follows:

#### Item generation

The aim was to provide a comprehensive list of all potential barriers for recruitment in our RCT. We generated potential questionnaire items based on the available literature, especially on the most recent systematic review of Fayter et al. [16] on recruitment barriers and on the "STEPS" study on strategies of trial enrollment by Campbell et al. [8]. Furthermore we incorporated all concerns and reasons of the involved physicians that were raised during the recruitment process as well as the suggestions proposed by the steering committee of the trial. We merged items that were deemed to be too similar to be assessed separately.

#### Item selection

**Figure 1:**
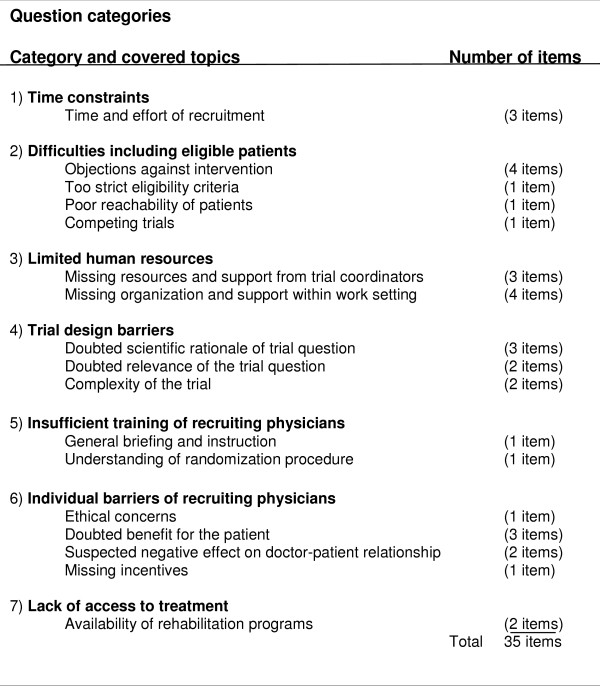
**Question categories**.

After item generation, the members of the steering committee selected those items that applied to our RCT and we included all of them in our questionnaire. These items covered the following categories (Figure 1): "Time constraints" (3 items); "Difficulties including eligible patients" (7 items); "Limited human resources" (7 items); "Trial design barriers" (7 items); "Insufficient training of recruiting physicians" (2 items); "Individual barriers of recruiting physicians" (7 items); And "lack of access to treatment" (2 items).

#### Development of questions and answer options

**Figure 2:**
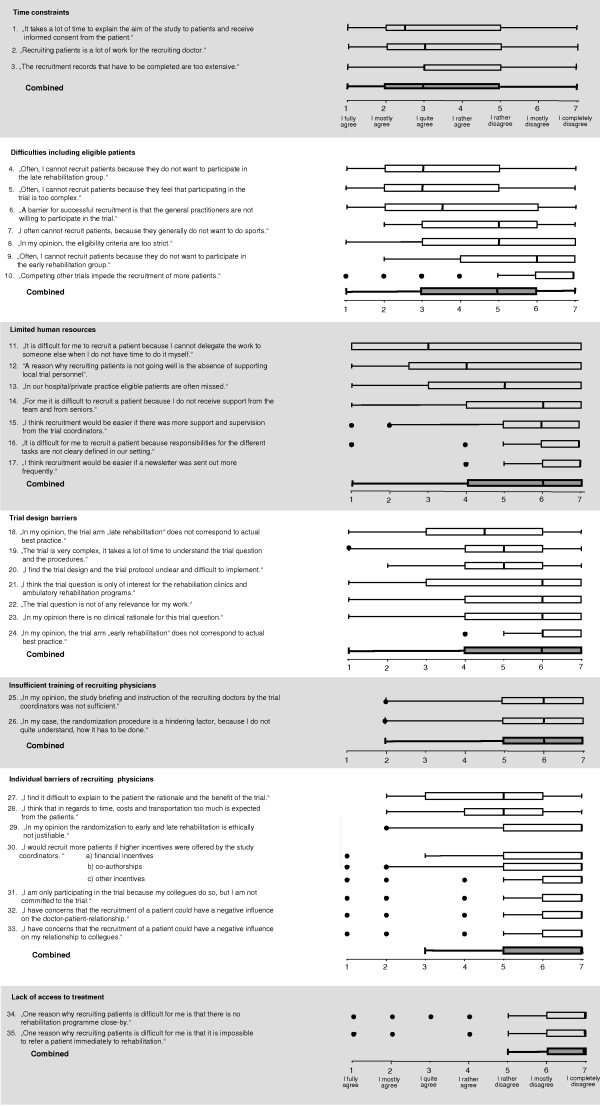
**Results of all 35 questionnaire items**. Statements about potential recruitment barriers were assigned to 7 categories. Physicians were asked to express their agreement with these statements on a Likert-type scale from 1 to 7. Box plots with medians and interquartile ranges summarize the answers of the physicians. For each category a summary plot of the answers is presented. Because of limited space some of the questions are slightly shortened. For the original question, please see the appended questionnaire.

For each of the selected items a separate question was developed and framed as a statement in German. For example, one item read as follows (English translation): "It is difficult for me to recruit a patient because I cannot delegate the work to someone else when I do not have time to do it myself". Participants then had to indicate how strong they agreed with the particular statement. We chose a Likert-type scale with 7 answer options ranging from "I fully agree" (1) to "I completely disagree" (7) (Figure 2). Lower scores indicated a greater recruitment barrier. The Likert-type scale offered respondents to express their agreement quantitatively rather than providing only a yes/no answer as it was done in earlier studies. Finally, we offered respondents to list and rate any additional statements describing recruitment barriers that were not included in the questionnaire. Figure 2 shows the 35 questions, the questionnaire is available online as well (see Additional file 1).

#### Pilot testing

After the development, we pilot tested the self-administered questionnaire with 6 physicians who did not participate in the survey to identify difficulties in understanding or missing items.

### Statistical analysis

For each item we calculated the median and the corresponding interquartile range (IQR) and displayed the results graphically using box plots. We combined the medians of single items within categories by calculating their median and IQR. All analyses were done with STATA for Windows version 10 (Stata Corp; College Station, TX).

## Results

38 out of 55 recruiting physicians returned the anonymised questionnaire (return rate 69%). 3 physicians did not answer any questions and returned a blank questionnaire, therefore, the useable response rate was 64%. We included the remaining 35 physicians in the analysis, whose questionnaires had few missing data (all items with <3%). 16 physicians (46%) were based at a hospital, 6 (17%) were in private practice and 12 (34%) were both at a hospital and in private practice (1 answer missing). The median age of the participants was 47.5 years (IQR 42.5 – 54.3) and most of them were specialists of both General Internal Medicine and Respiratory Medicine (23 physicians, 66%) whereas 6 physicians (17%) were specialists of Respiratory Medicine only. 22 physicians (63%) had been participating in the trial since the beginning of the trial and most of them had not yet recruited a patient at the time of the survey (23 physicians, 66%). The median number of eligible patients seen by recruiting physicians in the 3 months previous to the survey was 3 (IQR 1–5). The median percentage of eligible patients asked to participate, which also included informing the patients and asking for informed consent, was 75% (IQR 35–100%). Recruiting physicians reported that most patients identified to be eligible declined to participate (median 100%, IQR 90–100%).

### Overall result

Figure 2 shows the results for all 35 items as well as the summary scores for each category. Overall, "time constraints" (items combined median 3 [IQR 2–5]), represented the greatest barrier to trial recruitment followed by "Difficulties including eligible patients" (items combined median 5 [IQR 3–6]). All other problems were not regarded to represent substantial barriers to recruitment.

### Detailed results

The three questions addressing "time constraints" were answered homogenously (medians between IQR 2.5 and 3). The, on average, quite strong agreement with the three statements shows that limited time resources represent a substantial barrier to trial recruitment. However, IQR were quite wide and indicate that there is some variability in how physicians perceived time to be a critical factor for patient recruitment.

"Inclusion of eligible patients" is difficult if patients do not want to be randomized to late rehabilitation (median 3 [2-5]) or if trial participation is deemed to be too complex by patients (median 3 [IQR 2-5]). Another barrier represents the refusal of general practitioners, who see many exacerbated COPD patients, to participate in the recruitment process (median 3.5 [IQR 2-6]).

"Limited human resources" in the organization of recruiting physicians was, across all questions, not an important barrier. However, recruiting physicians appear not to get sufficient support from their team if they cannot do the recruitment process themselves (median 3 [IQR 1-7]). Also, local study personnel supporting recruitment would be appreciated by some recruiting physicians (median 4 [IQR 2.5–7]). But variability in responses to these questions was high again.

There was little heterogeneity across the questions about "trial design barriers". The only item that recruiting physicians reported to have some negative impact on recruitment was "In my opinion, the trial arm "late rehabilitation" does not correspond to actual best practice" (median 4.5 [IQR 3 -6]) In general, recruiting physicians felt to be trained adequately. Also, physicians did not agree with the statements about potential own barriers, which shows that ethical concerns, costs, incentives and the relationship with patients or colleagues were not significant barriers to recruitment. Finally, "lack of access to treatment" (respiratory rehabilitation) was not considered by physicians to have a negative impact on recruitment.

## Discussion

### Main findings

This survey suggests that time constraints and problems of enrolling eligible patients represent the greatest barrier for recruiting physicians of a multi-center RCT. Other barriers including organizational problems, trial design-related barriers, individual barriers and insufficient recruitment training appear to have little or no impact on recruitment.

### Strengths and weaknesses

Our survey had several strengths and limitations. First, this is to our knowledge the first survey on recruitment barriers that not only identifies recruitment barriers but also provides a weighting in respect to their actual impact on recruitment. Second, we tried to design and conduct a survey of high methodological quality following the recommendations of Fayter et al. [16] who identified a number of limitations in earlier surveys. For example, we provided reporting of the survey design, methods of data collection and analysis, we avoided selection bias by inviting all recruiting doctors to participate in the survey, we asked participants about any reasons for failure of recruitment instead of asking only specific aspects of patient recruitment, we provided respondents with the opportunity to make additional comments, and we asked physicians directly about recruitment barriers instead of asking for reasons why patients refused to participate.

A limitation is that we only focused on recruitment barriers from the physicians' perspective. We did not include the patients' perspective because we did not have any direct contact to patients before recruitment. We avoided asking physicians about recruitment barriers from the patients' perspective since this is likely to bias the results [15]. Also, using fully structured anonymised questionnaires (this is standardized questions as well as standardized answer options) offered no possibility to explore individual recruitment barriers in more depth. Unstructured interviews would have provided more insights into individual recruitment barriers. However, this would have come at the price of not allowing for quantitative analyses of the size of recruitment barriers as we did in the present study. Another limitation is that the response rate was 64%. We cannot rule out the possibility of selection bias with may have resulted in responses that are different from those who did not participate in the survey. Finally, although most of the questions addressed recruitment problems encountered in many trials, some of the questions included in the questionnaire were specific for our trial setting. This may impact the generalisability of some of our findings. On the other hand, recruiting physicians may provide better informed answers if the questions address problems that are directly applicable to their situation. Overall, we do not think that our findings apply to our trial setting only. Most physicians have substantial time constraints in their daily practice and study duties are likely to be of lower priority than clinical duties for most physicians.

### Recruitment barriers

Previous studies on recruitment problems in RCTs were conducted mainly in oncology and to a smaller extent in cardiovascular health and other disciplines. Also, most studies focused on recruitment problems from the patients' perspective whereas the physicians' perspective has been considered less frequently. How do the results from our survey compare to the results of previous studies? Surveys from the physicians' perspective in other clinical settings (mainly oncology) also identified time constraints, including time required to obtain informed consent [16,3] and difficulties including eligible patients [16] as a major barrier for patient recruitment. Also similar to our results, lack of incentives [3], trial design barriers [16,3], limited human resources and insufficient training of recruiting physicians [3] were only reported by a few studies to represent major barriers.

The identification of the most important recruitment barriers in the planning of a RCT is undoubtedly an important prerequisite to assess the feasibility of a trial [16]. Still, it is important to note that a successful recruitment also depends much on the motivation of recruiting physicians for the study and how they communicate the study to their patients [22]. We did not ask physicians in detail about their motivation to participate and their understanding of the trial design because we did not think that a postal survey would yield valid insights in regard to these aspects. Although there are some clues in literature regarding the importance of these factors [23], there is a need to further evaluate the impact of these more complex factors on recruitment success.

### Interventions to minimize recruitment barriers

We found that major barriers for trial recruitment were time constraints and the fact that eligible patients could not be included in the trial for several reasons. Other factors appear to have almost no impact on recruitment, at least from the physicians' perspective. These findings have important implications for deciding about measures to minimize recruitment barriers.

What is the evidence for the effectiveness of interventions aimed at minimizing recruitment barriers from the physicians' perspective? A systematic review [23] focused on interventions to overcome barriers for clinicians to participate in trials. No randomized trials were identified but only observational studies that did not support any of the interventions. Campbell et al. conducted an analysis of 114 trials and analyzed potentially influential factors on trial recruitment[8]. However, they did not explicitly evaluate interventions to improve trial recruitment. Further systematic reviews [4,17,18] focused primarily on interventions to overcome barriers to patient participation. Thus it appears that most research on recruitment barriers and on interventions to address these barriers have focused on the patients' perspective.

To our knowledge, there are no randomized trials evaluating the effectiveness of interventions to minimize recruitment barriers from the physicians' perspective. This is surprising as many trials, especially multi-centre trials, experience recruitment problems with physicians rather than with patients [23]. Based on our results, the most promising approach may be to address time constraints of recruiting physicians. Obviously, providing sufficient study personnel might be the most effective intervention. But this might only be an option for industry-sponsored trials with sufficient funding. For investigator-driven trials alternatives are needed. A possible intervention is, for example, to provide a study nurse in a certain catchment area who regularly contacts participating physicians by emails or phone calls and assists with time consuming recruitment activities. Also offering co-authorship or moderate financial incentives to recruiting physicians may be effective strategies to motivate them to spend more time with research activities. Neither our trial nor previous studies provide valid estimates about the effects of these measures to minimize recruitment barriers. The most adequate way to test their impact on recruitment remains a randomized trial that could be incorporated into a randomized treatment trial.

## Conclusion

From the recruiting physicians' perspective time constraints and difficulties including patients identified to be eligible appear to have the largest impact on recruitment in randomized trials. Effective but also affordable measures to minimize these important recruitment barriers should be developed and evaluated.

## Competing interests

The authors declare that they have no competing interests.

## Authors' contributions

All authors conceived the study idea; MP and AS designed the study; AS collected the data; MP and AS analysed the data and drafted the manuscript; All authors revised the manuscript and approved the final version of the submitted publication.

## Pre-publication history

The pre-publication history for this paper can be accessed here:

http://www.biomedcentral.com/1471-2288/9/14/prepub

## Supplementary Material

Additional file 1 **Survey questionnaire**. SOPRE study, Barriers to involvement in the study Click here for file
