# Supplementary material for: Recruitment barriers in a randomized controlled trial from the physicians' perspective – A postal survey
Source: BMC Med Res Methodol. 2009 Mar 2;9:14. doi: 10.1186/1471-2288-9-14 (PMC2653070; doi:10.1186/1471-2288-9-14)
Supplement: Additional file 1 — Survey questionnaire. SOPRE study, Barriers to involvement in the study [file 1471-2288-9-14-S1.pdf]

SOPRE Study
Barriers to involvement in the study

Dear colleagues,

We would be grateful if you could complete the following questionnaire.
The aim of the questionnaire is to determine the reasons for the difficulty in recruitment to the SOPRE study and thereby make improvements possible.

Please put a cross in only one answer per question and return the questionnaire in the enclosed envelope within 2 weeks.

We would like to thank you very much for your participation and feedback.

The SOPRE study team

Basic data on the physician completing the form

Today's date ___/___/_____ (DD/MM/YYYY)

I. Age in years _______ (YY)

II. Function in hospital ☐ practice ☐

III. FMH title Internal Medicine ☐ Pneumology ☐
Other ☐ None ☐

IV. Year of final examination _______ (YYYY)

V. When were you briefed about recruitment to the SOPRE study?
(Only one answer possible)

Participated from the ontset (September 2006) ☐
Within the last 6-12 months ☐
Within the last 6 months ☐

VI. Have you already recruited a patient?

Yes ☐ No ☐

VII. How many patients complied with the inclusion criteria during the **last 3 months**?

_______ (estimated number)

How many of these (in percent) did you ask about participation in the study?
Approximately

_______%

How many of these (in percent) refused to participate in the study?
Approximately

_______%

Please indicate in each case how strongly you agree with the statements	Agree completely **1**	Agree largely **2**	Agree mostly **3**	Agree moderately **4**	Disagree moderately **5**	Disagree largely **6**	Disagree completely **7**
**A. System-related and organisational problems**							
**A.I. Time factor**							
1. "The recruitment records, that have to be completed, are too extensive. "	1	2	3	4	5	6	7
2. "It takes a lot of time to explain the aim of the study to patients and obtain Informed Consent. "	1	2	3	4	5	6	7
3. "In my opinion recruitment creates too much work for the doctors involved. "	1	2	3	4	5	6	7
**A.II. Disagreement within the team/hospital**							
1. „I find recruitment difficult because I do not receive any support within the team or from senior staff. "	1	2	3	4	5	6	7
**A.III. Organisation and resources**							
**a) Within the hospital/practice**							
1. "I find recruitment difficult because responsibilities for the individual recruitment tasks are not clearly defined within our organisation. "	1	2	3	4	5	6	7
2. "I find recruitment difficult because I cannot delegate the work if I do not have time for it myself. "	1	2	3	4	5	6	7
3. "In our hospital/practice the opportunity to involve suitable patients is frequently missed. "	1	2	3	4	5	6	7
**b) Within the trial**							
4. "In my opinion recruitment would be easier if there was more support and supervision by the trial coordinators. "	1	2	3	4	5	6	7
5. "I think recruitment would be easier if a newsletter was sent out more frequently. "	1	2	3	4	5	6	7

Please indicate in each case how strongly you agree with the statements	Agree completely **1**	Agree largely **2**	Agree mostly **3**	Agree moderately **4**	Disagree moderately **5**	Disagree largely **6**	Disagree completely **7**
6. "One reason why recruitment does not run well is the absence of supporting local study personnel. "	1	2	3	4	5	6	7
**A.IV. Briefing and communication on the study**							
1. "In my opinion the briefing and training for recruiters provided by the trial coordinators was not sufficient. "	1	2	3	4	5	6	7
2. "I find the randomization procedure an obstacle as I am not absolutely certain how it has to be done. "	1	2	3	4	5	6	7
**A.V. Identification of study patients**							
1. "In my case competing studies prevent me from recruiting more patients. "	1	2	3	4	5	6	7
2. "In my opinion the inclusion criteria are too strict. "	1	2	3	4	5	6	7
3. "One obstacle to successful recruitment is that the general practitioners, who look after most of the suitable patients, are not taking part. "	1	2	3	4	5	6	7
4. "I am often unable to recruit patients because they do not want to do any sport as a matter of principle. "	1	2	3	4	5	6	7
5. "I am often unable to recruit patients because they do not want to participate in the late rehabilitation group. "	1	2	3	4	5	6	7
6. "I am often unable to recruit patients because they do not want to participate in the early rehabilitation group. "	1	2	3	4	5	6	7
7. "I am often unable to recruit patients because they feel that participating in the trial is too time-consuming. "	1	2	3	4	5	6	7

Please indicate in each case how strongly you agree with the statements	Agree completely **1**	Agree largely **2**	Agree mostly **3**	Agree moderately **4**	Disagree moderately **5**	Disagree largely **6**	Disagree completely **7**
**A.VI. Structural problems**							
1. "One reason why recruiting patients is difficult for me is that there is no rehabilitation programme close-by. "	1	2	3	4	5	6	7
2. "One reason why I find recruitment difficult is that I am unable to refer a patient immediately to rehabilitation. "	1	2	3	4	5	6	7
**B. Problems related to the study design**							
**B.I. Study question**							
1. "The trial question is not relevant for my work. "	1	2	3	4	5	6	7
2. "In my opinion there is no clinical rationale for this trial question. "	1	2	3	4	5	6	7
3. "In my opinion the trial arm "late rehabilitation" does not correspond to current best practice. "	1	2	3	4	5	6	7
4. "In my opinion the study arm "early rehabilitation" does not correspond to current best practice. "	1	2	3	4	5	6	7
5. "In think the trial question is only of interest for the rehabilitation clinics and the outpatient rehabilitation programmes. "	1	2	3	4	5	6	7
**B.II. Study protocol**							
1. "The trial is very complex and requires a lot of time in order to understand the trial question and the approach. "	1	2	3	4	5	6	7
2. "I find the trial design and the trial protocol unclear and difficult to implement. "	1	2	3	4	5	6	7

Please indicate in each case how strongly you agree with the statements	Agree completely **1**	Agree largely **2**	Agree mostly **3**	Agree moderately **4**	Disagree moderately **5**	Disagree largely **6**	Disagree completely **7**
**C. Individual barriers of recruiting physicians**							
**C.I. Ethical concerns**							
1. "In my opinion, the randomization to early and late rehabilitation is ethically not justifiable. "	1	2	3	4	5	6	7
**C.II. Benefit of the study for patients**							
1. "I have difficulties in explaining the rationale and benefit of the trial to the patient. "	1	2	3	4	5	6	7
2. "In my opinion too much is expected of the patient in terms of time, costs and transport/distance. "	1	2	3	4	5	6	7
3. "I only agreed to participate because other colleagues are also involved, but I do not support the study. "	1	2	3	4	5	6	7
**C.III. Doctor-patient relationship**							
1. "I have concerns that the recruitment of a patient might have a negative influence on the doctor-patient relationship. "	1	2	3	4	5	6	7
2. "I have concerns that the recruitment of a patient might have a negative influence on my relationship with my colleagues (e.g. general practitioners). "	1	2	3	4	5	6	7
**C.IV. Lack of incentives to participate**							
1. "I would be more inclined to recruit patients if more attractive incentives incentives were offered by the study coordinators. "							
a) Regarding financial compensation	1	2	3	4	5	6	7
b) Regarding co-authorships	1	2	3	4	5	6	7
c) Regarding other issues_________________(please indicate)	1	2	3	4	5	6	7

D. Other factors

D.I. Comments on the questions listed above (please indicate the number of the question in each case)

____

____

____

____

D.II. Further reasons for the difficulty in recruitment which have not yet been mentioned (Please indicate a weighting for every topic which you mention.)

Please indicate in each case how strongly you agree with the statements	Agree completely **1**	Agree largely **2**	Agree mostly **3**	Agree moderately **4**	Disagree moderately **5**	Disagree largely **6**	Disagree completely **7**
1.________________________________	1	2	3	4	5	6	7
2.________________________________	1	2	3	4	5	6	7
3.________________________________	1	2	3	4	5	6	7
4.________________________________	1	2	3	4	5	6	7

Many thanks for your participation

Horten-Center University of Zurich P.O. Box Nord 8091 Zurich Tel (044) 255 87 10 anne.spaar@usz.ch
